# Clinical presentation of perineal endometriosis and prognostic nomogram after surgical resection

**DOI:** 10.1186/s12905-022-02068-3

**Published:** 2022-11-26

**Authors:** Shiyang Zhu, Zhiyue Gu, Xiaoyan Li, Yi Dai, Jinghua Shi, Jinhua Leng

**Affiliations:** 1 Department of Obstetrics and Gynecology, Peking Union Medical College Hospital, Chinese Academy of Medical Sciences and Peking Union Medical College, No. 1 Shuaifuyuan No. 1, Dongcheng District, Beijing, 100730 China; 2 National Clinical Research Center for Obstetric and Gynecologic Diseases, Beijing, China; 3 Department of Gynecology and Obstetrics, West China Second University Hospital, Sichuan University, Chengdu, China

**Keywords:** Perineal endometriosis, Anal sphincter involvement, Prognostic model, Nomogram, Recurrence

## Abstract

**Background:**

This retrospective study evaluated the clinical features of perineal endometriosis (PEM) and established a prognostic nomogram for recurrence probability in patients treated with surgical resection.

**Methods:**

This study enrolled 130 PEM patients who had received surgical treatment in Peking Union Medical College Hospital (PUMCH) between January 1992 and September 2020. We collected their clinical features and conducted outpatient or telephone follow-up. The predictive nomogram was constructed based on 104 patients who had completed follow-up by July 2021. The Cox proportional hazards regression model was used to evaluate the prognostic effects of multiple clinical parameters on recurrence. The Index of concordance (C-index) and calibration curves were used to access the discrimination ability and predictive accuracy of the nomogram respectively, and the results were further validated via bootstrap resampling. Calculating the area under the curve (AUC) via risk scores of patients aimed to further access the predictive power of the model. In addition, the survival curve was depicted using Kaplan–Meier plot and compared by log-rank method.

**Results:**

Most PEM patients had been symptomatic for 24–48 months before the lesion resection. With a median 99.00 (interquartile range: 47.25–137.50) months of postoperative observation, there were 16 (15.1%) out of 104 cases who finished follow-up reported symptomatic recurrence. On multivariate analysis of derivation cohort, multiple lesions, microscopically positive margin (mPM) and anal sphincter involvement (ASI) were selected into the nomogram. The C-index of the nomogram for predicting recurrence was 0.84 (95% CI 0.77–0.91). The calibration curve for probability of recurrence for 36, 60 and 120 months showed great agreement between prediction by nomogram and actual observation. Furthermore, the AUCs of risk score for 36, 60 and 120 months were 0.89, 0.87 and 0.82 respectively.

**Conclusions:**

PEM is a rare kind of endometriosis and surgery is the primary treatment. Multiple lesions and ASI are independent risk factors for postoperative recurrence, and wide resection with more peripheral tissue could be preferred. The proposed nomogram resulted in effective prognostic prediction for PEM patients receiving surgical excision. In addition, this predictive nomogram needs external data sets to further validate its prognostic accuracy in the future.

## Background

Endometriosis is a chronic inflammatory disease characterised as the presence of endometrial tissue outside of the uterine cavity, which affects 6% to 10% of women of reproductive age [1]. Pelvic lesions at peritoneal surface, ovaries, and uterus ligaments are the most common locations of endometriosis, other organs such as intestine, ureter, thorax, even nasal cavity can also been involved [2]. Perineal endometriosis (PEM) is a rare type that accounts for merely 0.17% to 0.37% of women treated for endometriosis [3, 4]. Characterised by the ectopic endometrial tissue located in the subcutaneous adipose layer of perineum, PEM is predominantly associated with injuries caused by episiotomy or obstetrical tears [5–7]. Patients with PEM typically show solid tender nodule or mass with cyclic pain around the perineal scar [6]. Moreover, the infiltrative growth of lesions may lead to an increased risk of involvement with adjacent structures such as anal sphincter, vaginal wall, or rectum [4, 5].

Diagnosis and treatment for PEM can still be very difficult currently, mostly due to its high rarity and consequently low awareness among clinicians. A systematic review suggests that there have been only around three hundreds PEM cases documented since 1923 all over the world [5]. Over the centenary, progress has been made in the knowledge about PEM, while timely diagnosis and treatment can still be restricted because most patients complaining of perianal pain are primarily treated by non-gynaecological clinicians. Given the lack of integrity and consistency in current data, we retrospectively collected consecutive series of 130 PEM patients who received surgical treatment in Peking Union Medical College Hospital (PUMCH) during the past nearly three decades, aiming to summarise the clinical features and treatment of PEM. We conducted outpatient or telephone follow-up on the prognosis of patients after surgical resection and developed a predictive nomogram.

Currently, nomograms have been widely developed in a variety of diseases [8]. As a graphical representation of a mathematical model, nomogram predicts a concerning event through clinical characteristics. By integrating various essential factors, a nomogram can estimate the probability of an individual developing a certain disease and the corresponding survival and recurrent rates after treatment [9]. The application of the nomogram model allows easier and faster prediction of outcomes in clinical practise. Therefore, besides presenting the clinical information of PEM patients, a prognostic nomogram for PEM patients was established for the first time according to the pre-surgical history and post-surgical follow-up information to further explore the rare disease.

## Methods

### Patients and materials

**Fig. 1:**
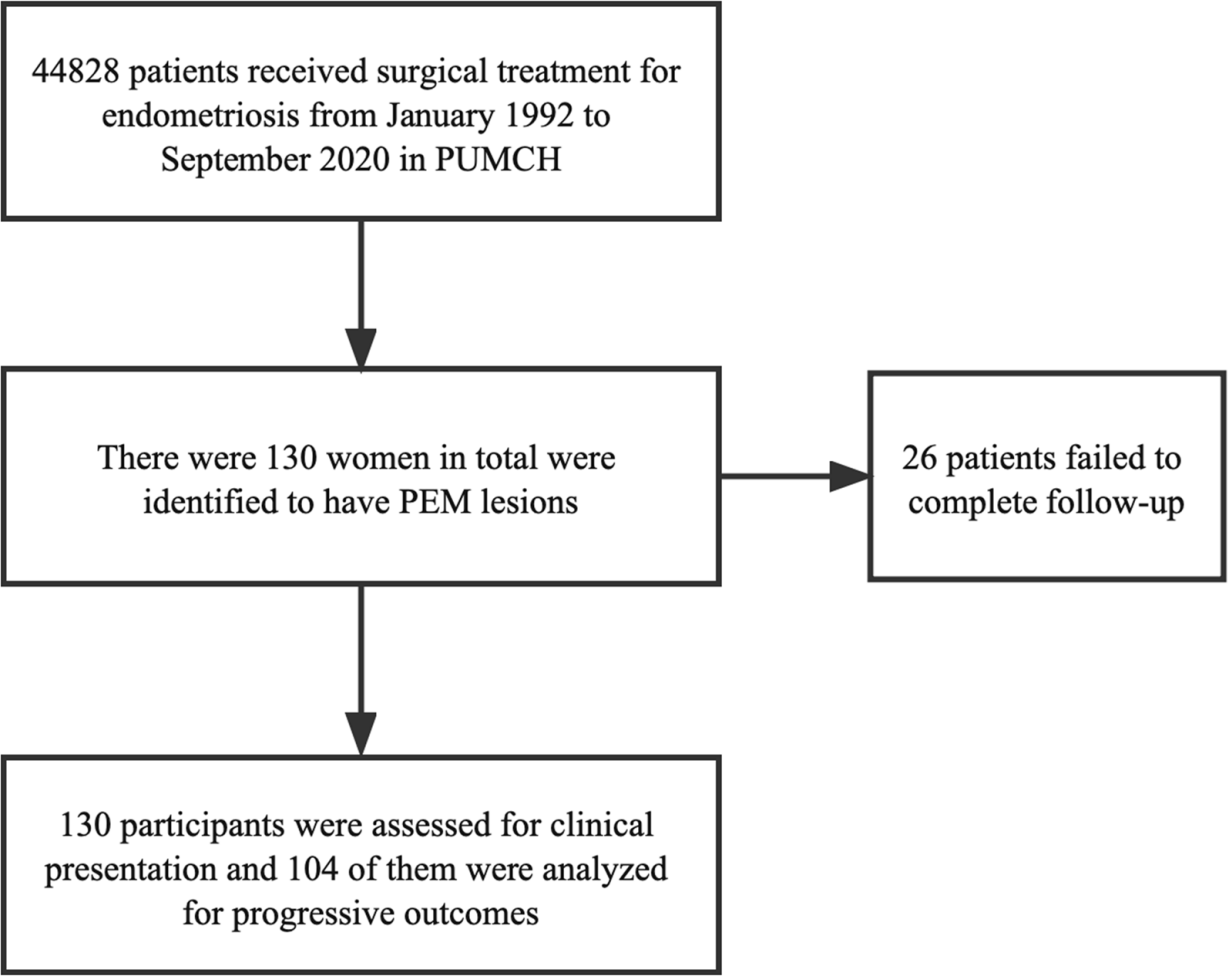
Flow diagram of included patients

The predictive study was conducted through a computer search of pathological records between January 1992 and September 2020 in PUMCH. Patients who had surgical removal of PEM lesions with histologically confirmed diagnosis were included. The demographic data, medical history, clinical symptoms, imaging results, pharmacological treatment before and after the surgery and postoperative complications were all collected. This study was approved by the Ethics Committee of PUMCH (JS-1722). Written informed consent was obtained from all participants. The flow diagram of included patients is shown in Fig. 1.

We carried out preoperative assessment for patients. The serum levels of cancer antigen 125 (CA-125) and ultrasonographic examination were the two most common examinations for pre-surgical evaluation. Detailed screening by transvaginal ultrasound (TVS) was conducted to exclude potential pelvic abnormalities including pelvic endometriosis and adenomyosis. Thorough physical examination including the bimanual pelvic examination and vagino-recto-abdominal examination was routinely performed on all patients. If the lesion was near vagina or anal canal, additional trans-perineal ultrasonography, endoanal ultrasonography or magnetic resonance imaging (MRI) should be performed.

**Fig. 2:**
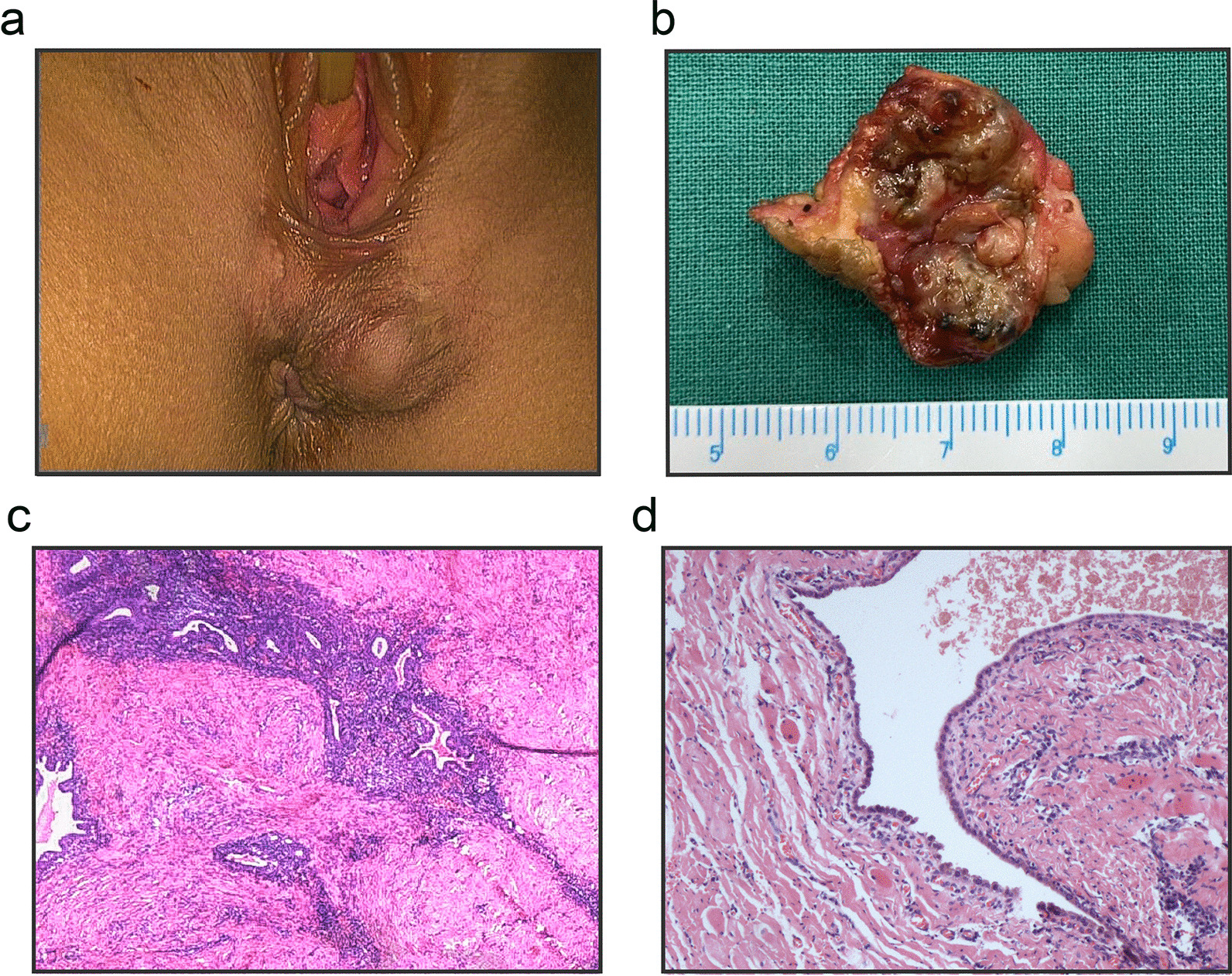
Histopathological presentations of perineal endometriosis (PEM) **A**: palpable nodules located in episiotomy scar of perineum; **B**: the gross specimen of PEM after surgical excision, components of old hemorrhage was observed; **C** and **D**: microscopic appearance of PEM with anal sphincter involvement, showing an endometrial gland appeared in the sphincteric muscular tissue (H&E stain, × 100)

**Fig. 3:**
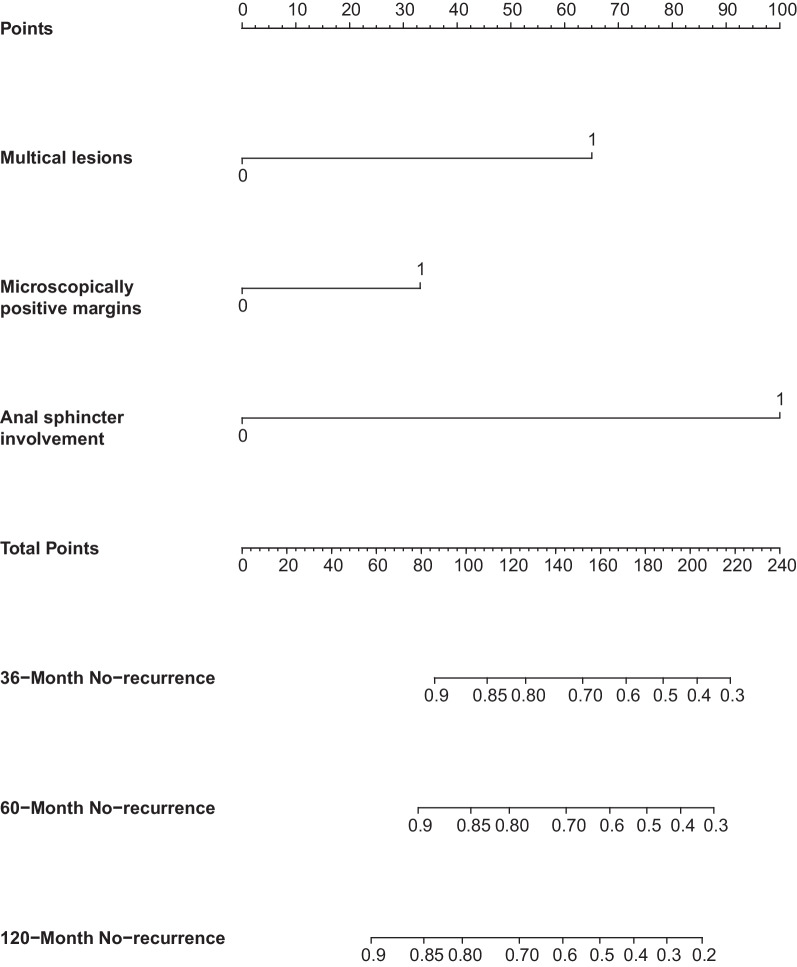
Nomogram for predicting the probability of 36-, 60- and 120 months PEM No-recurrence. To use the nomogram, draw a vertical line from each variable to the corresponding points scale to acquire its score, calculate the sum of all the scores, and draw a vertical line from the total points scale to the 36-, 60- and 120- months axis to obtain the probability of PEM No-recurrence. PEM, perineal endometriosis

The diagnosis of PEM was established when the patient was in presence of (1) menstruation-related pain at perineum, (2) increasingly enlarged nodules around the episiotomy scar (Fig. 2A and B), and (3) the surgical pathology showed typical endometrial gland or interstitial tissue in the resected specimen as well as excluding malignancy (Fig. 2C). As shown in Fig. 2D, the involvement of anal sphincter was determined when endometrial gland or stroma was found in the corresponding resected muscular tissues under microscopic examination.

The postoperative follow-up visit was conducted at out-patient clinic or by telephone. During follow-up period, patients were asked whether the painful symptom at perineum diminished or reappeared, and careful palpation around surgical wound including rectal and vaginal examination was performed in out-patient clinic. Trans-perineal ultrasonography was indicated when patients reported re-occurrence of menstruation-associated pain at the excisional site of previous surgery with or without palpable nodules. Recurrence was defined as the reoccurrence of cyclic pain at perineal scar with or without coexisting PEM lesions under ultrasonographic scanning. Given the long duration of follow-up, the observation ended up after the patients experienced spontaneous menopause.

On the basis of the recurrent outcomes, participants who accomplished the final visit in July 2021 and post-menopausal cases with definitive outcome were divided into recurrent group and non-recurrent group. Post-operative information including related symptoms, imaging results, recurrence time and post-recurrence treatment was collected.

### Statistical methods

Continuous variables were present as mean ± standard deviation (SD) or median with interquartile range (IQR), and categorical variables were showed as percentage. Univariate and multivariate analyses of Cox regression model were cooperated to explore recurrence related factors. Statistical analysis was performed using SPSS Version 22.0 (SPSS Inc., Chicago, IL, USA), and a nomogram was established using R 4.0.0 software according to the results of multivariate analysis (Institute for Statistics and Mathematics, Vienna, Austria; http:// www.r-project.org/). More information on screening of recurrence related factors, nomogram validation (bootstrap resampling method and calibration curve), the assessment of predictive accuracy (via the area under the curve, AUC), and the depiction and comparation of survival curves (via Kaplan–Meier method and log-rank test, separately) were referred to the previously published article [10].

## Results

### The demographic and clinical information of 130 PEM patients

The demographic and clinical information of 130 PEM patients is shown in the Table 1.

**Table 1:** The demographic and clinical information of 130 PEM^a^ patients

Variable	Patients number	Mean ± SD^b^, Median (IQR^c^) or Percentage
Demographic data		
Age at surgery, yr	130	33.40 ± 4.55
Height, cm		
Weight, kg		
BMI^d^, kg/m^2^	130	21.80 (20.15–23.75)
Gravidy	130	2 (1–2)
Parity	130	1 (1–1)
Age at delivery, yr	129	26.45 ± 3.45
Age at onset of symptoms		
Repeated surgery for PEM	28/130	21.5%
Clinical data		
Pathogenesis		
Spontaneous	1/130	0.8%
Episiotomy	99/130	76.2%
Obstetrical lacerations	29/130	22.3%
Episiotomy & Obstetrical laceration	1/130	0.8%
Latent period, mo	119	36.00 (12.00–54.00)
Duration of symptoms, mo	130	36.00 (24.00–48.00)
CA^e^ 125, IU/L	100	22.15 (14.38–33.46)
CA 125 ≥ 35 IU/L	20/100	20.0%
Dysmenorrhea	33/130	25.4%
Coexistent OEM^f^	15/130	11.5%
Coexistent AM^g^	18/130	13.8%
Preoperative GnRH-a^h^	78/130	60.0%
Surgical and pathological data		
Multiple lesions	32/130	24.6%
Size of lesions, cm	130	2.50 (2.00–3.00)
Size of lesions ≥ 3 cm	55/130	42.3%
Anal sphincter involvement by pathology	43/130	33.1%
Positive cut edge	37/120	30.8%
Location of the lesion		
Middle	19/120	15.8%
Left side	92/120	76.7%
Right side	9/120	7.5%
Postoperative data		
Postoperative complications	17/130	13.1%
Delayed healing	12/130	9.2%
Wound infection	2/130	1.5%
Fistula	2/130	1.5%
Uroschesis	1/130	0.8%
Hospitalization days	126	9 (7–12)
Postsurgical medication		
None	70/130	53.8%
GnRH-a	36/130	27.7%
Progestogen	13/130	10.0%
COCs^i^	11/130	8.5%
Recurrence	16/104	13.3%

^a^*PEM* perineal endometriosis; ^b^*SD* standard deviation; ^c^*IQR* interquartile range; ^d^*BMI* body mass index; ^e^*CA-125* cancer antigen 125; ^f^*OEM* ovarian endometriosis; ^g^*AM* adenomyosis; ^h^*GnRH-a* gonadotrophin releasing hormone agonist; ^i^*COCs* combined oral contraceptives

With mean age of 33.40 ± 4.55 (range: 21–50) years at surgery, 129 patients had undergone perineal trauma due to episiotomy or obstetrical lacerations and only 1 developed PEM lesion spontaneously. Following the spontaneous delivery, the lesion took a median of 36.00 (IQR: 12.00–54.00) months to develop. Participants usually did not get motivated to search for medical help until the aggravating pain became unbearable, which took a median of 36.00 (IQR: 24.00–48.00) months after the onset of symptoms.

The results showed that twenty (20/100, 20.0%) patients demonstrated elevation in serum CA-125 levels ranging from 35.5 to 122.4 IU/L. Fifteen (15/130, 11.5%) patients had cooccurrence of ovarian endometrioma and eighteen (18/130, 13.8%) adenomyosis according to medical history and imaging examination. With a median level of 22.15 (IQR: 14.38–33.46) IU/L for all 100 participants with available CA125 results, it seemed that the serum CA125 did not assist the diagnosis of PEM.

The gonadotrophin-releasing hormone agonist (GnRH-a) had been administrated in 78 (78/130, 60.0%) patients preceding the surgery aiming to reduce the size of lesion and finally narrow the surgical defect whereby the lesion significantly shrunk from 2.47 ± 0.62 cm to 1.91 ± 0.57 cm (*P* < 0.001) in greatest dimension according to 29 patients with ultrasonographic measurement before and after the treatment.

Among the 130 patients, 129 (99.2%) received local narrow excision with 0.3–0.5 cm of surgical margin under general anaesthesia, only 1 (0.8%) had incomplete excision due to the extensive involvement with anal sphincter and rectum. The median size of excised PEM lesion was 2.50 (IQR: 2.00–3.00) cm. Out of 130 patients, 32 (24.6%) women had multiple lesions, and a total of 165 lesions were resected. Most lesions were multilocular cysts containing characteristic blood content (Fig. 2B).

It should be noted that the lesions of 43 patients (43/130, 33.1%) were histologically confirmed to involve the external anal sphincter. Additionally, 12 (12/130, 9.2%) affected women with concomitant ovarian cyst received laparoscopic cystectomy as well, which turned out to be ovarian endometrioma (10/130, 7.7%) and teratoma (2/130, 1.5%).

Empirically, postoperative treatment was applied regarding the age at surgery, lesion size, sphincter invasion and whether it was complicated with pelvic endometriosis. Medical treatments including GnRH-a, combined oral contraceptives and progestins (namely dienogest, gestrinone, levonorgestrel, hydroxyprogesterone caproate and medroxyprogesterone acetate) were offered to 60 (60/130, 46.2%) cases after surgery. Meanwhile, 9 (9/130, 6.9%) of these 60 women received combined insertion of levonorgestrel-releasing intra-uterine system (LNG-IUS) for the treatment of coexistent adenomyosis after the systemic hormone treatment mentioned above.

### Clinical information and risk factors of recurrent patients

The therapeutic outcomes were available in 104 cases since 26 participants were lost during follow-up. There were 15 women had definitive clinical outcomes as they experienced spontaneous menopause during the long-term observation.

Menstruation associated pain and palpable mass at perineal area reshowed in 16 (15.4%) of 104 patients during a median 99.00 (IQR: 47.25–137.50) months of follow-up observation, including the one with incomplete resection. The mean age of 16 patients with recurrence was 32.75 ± 3.70 (range: 25–39) years at surgery. Five (5/16, 31.25%) cases had repeated local lesion excision with histopathologic confirmation of recurrence. One case (Case No.2) exhibited extensive anal invasion along with a surge in serum levels of CA-125 (151 IU/L) and CA 19–9 (44.1 IU/L) on recurrence. Biopsy of the lesion ruled out malignant changes and confirmed the recurrence of PEM. GnRH-a was subsequently applied for 4 months but with no response. Eventually, the patient received intravaginal radiotherapy, after which the lesion regressed whereas ovarian failure induced by the radiotherapy took place concomitantly. Case No.1 accepted bilateral salpingo-oophorectomy and hysterectomy because of the severe dysmenorrhoea caused by coexistent adenomyosis, thereafter the painful symptom diminished. The remaining nine (9/16, 56.25%) patients with clinical recurrence received medical treatment as long-term management because they refused to have additional surgeries. Up to present, the medical therapy has worked on well in these participants as the periodic pain at perineum has been relieved and the lesion size has stabilised. The demographic and clinical information of 16 patients with recurrent PEM is shown in Table 2.

**Table 2:** Clinical information and treatment of 16 patients with recurrent PEM^a^

Recurrent cases, No	Number of lesions	Size of lesions, cm	ASI^b^	Preoperative GnRH-a^c^	Postoperative Hormone therapy	Disease-free interval, mo	Size of recurrent lesion, cm	Treatment for recurrence
1	2	2.0; 1.5			MPA^d^	72	2	Bilateral oophorectomy and total hysterectomy
2	1	2.5		Y	GnRH-a	96	3	Intravaginal radiation
3	2	5.0; 1.0	Y		GnRH-a	3	3	Surgical excision, GnRH-a
4	1	2.5				30	3	Surgical excision, GnRH-a
5	1	2	Y	Y	Gestrinone	3	2.5	Surgical excision, GnRH-a
6	2	3.5; 2.5	Y	Y	GnRH-a	60	1.5	GnRH-a, COCs^e^
7	2	4.0; 1.0	Y	Y	GnRH-a	15	2	LNG-IUS^f^
8	2	2.0; 1.0		Y	COCs	24	1.5	GnRH-a, COCs
9	2	3.0; 1.5	Y	Y		36	2.0, rectum involved	Surgical excision, LNG-IUS
10	1	2.5				99	1.3	COCs
11	2	2.0; 1.0	Y	Y	LNG-IUS	6	3	GnRH-a, COCs, LNG-IUS
12	2	2.5; 1.0			GnRH-a	4	1	TCM^g^
13	1	3	Y	Y	GnRH-a	12	1.5	COCs
14	2	3.0; 2.0	Y	Y		12	2	COCs
15	1	3	Y	Y	GnRH-a	6	1	LNG-IUS
16	2	2.0; 0.5		Y	GnRH-a	26	3	Surgical excision, GnRH-a

^a^*PEM* perineal endometriosis; ^b^*ASI* anal sphincter involvement; ^c^*GnRH-a* gonadotrophin-releasing hormone agonist; ^d^*MPA* medroxyprogesterone acetate; ^e^*COCs* combined oral contraceptives; ^f^*LNG-IUS* levonorgestrel-releasing intra-uterine system; ^g^*TCM*: traditional chinese medicine

During the univariate Cox regression analysis, the following variables were selected for further multifactor regression analysis: (1) *P* value < 0.1, such as “multiple lesions” (*P* < 0.001) and “anal sphincter involvement (ASI)” (*P* < 0.001); (2) although the *P* > 0.1, “microscopically positive margin (mPM)” (*P* = 0.102) was considered as important factor included for further multivariate analysis. Finally, three variables were included into the multivariate Cox regression analysis: multiple lesions, ASI and mPM. Furthermore, multivariate analysis determined that the multiple lesions (HR = 3.322, 95% CI: 1.1509–9.592, *P* = 0.026) and ASI (HR = 1.8458, 95% CI: 0.6711–2.750, *P* = 0.006) were independent risk factors for recurrence.

### Prognostic nomogram for whole recurrence

In the present cohort, three factors of the multivariate Cox regression model were included in the prognostic nomogram, which established scoring criteria in terms of the hazard ratio (HR) values of these factors. The prognostic nomogram for 36, 60 and 120 months without recurrence is shown in Fig. 3. By adding the scores corresponding to each variable and projecting the total score to the bottom scale, the probabilities of no recurrence for 36, 60 and 120 months could be estimated. The details about interpretation of nomogram could be referred to reported literature [10].

**Fig. 4:**
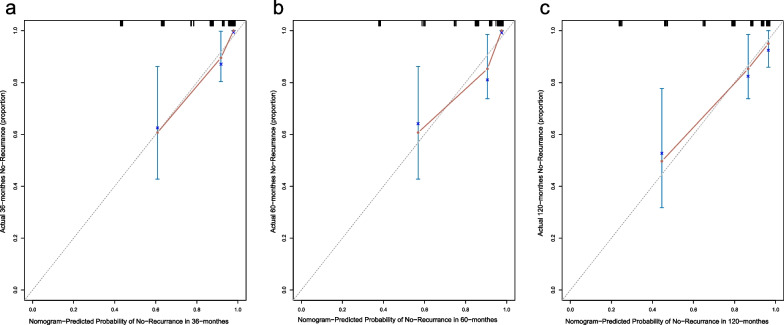
The calibration curve for predicting patients without recurrence at **A** 36-month, **B** 60-month and **C** 120-monthin the derivation cohort. Nomogram-predicted probability of overall without recurrence is plotted on the x-axis, actual overall without recurrence is plotted on the y-axis. The grey line represents a perfect fit between the nomogram predicted probability and the observed probability. The blue line represents performance of the present nomogram. Closer distances between the two lines represent higher prediction accuracy

**Fig. 5:**
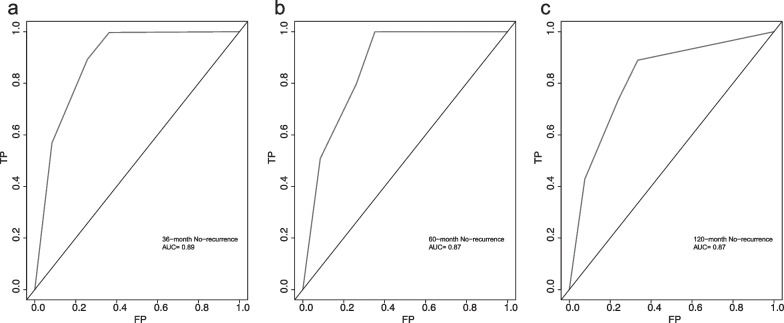
The area under curve (AUC) of nomogram predictive model at **A** 36-month (AUC = 0.89), **B** 60-month (AUC = 0.87) and **C** 120-month (AUC = 0.82) in the derivation cohort. The receiver ROC is made via R package “survivalROC”. AUC, area under curve; ROC, operating characteristic curve; TP, true positive-rate; FP, false positive-rate

**Fig. 6:**
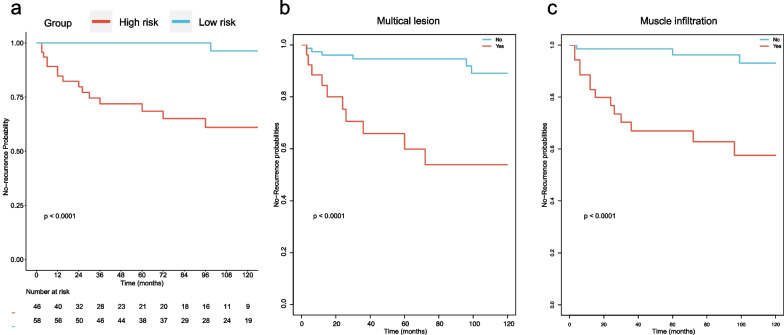
Kaplan–Meier survival curves of derivation cohort. The Kaplan–Meier method showed that there was a statistically significant difference in recurrence probability between **A** the low-risk group and the high-risk group (*P* < 0.001), **B** whether multiple lesions (*P* < 0.001) and **C** whether anal sphincter involvement (*P* < 0.001)

We further carried out the validation of Nomogram. The overall performance of the nomogram was assessed, producing a C-index of 0.84 (95% CI 0.77–0.91). The calibration curve revealed a desirable agreement between the predicted and observed values for 36, 60 and 120 months respectively (Fig. 4A, B and C). Furthermore, according to the risk scores calculated by Cox regression model and the observed recurrence results, the AUC for 36, 60 and 120 months without recurrence were 0.89, 0.87 and 0.82 respectively (Fig. 5A, B and C). In addition, the low-risk group and the high-risk group had a statistical difference in recurrence probability according to Kaplan–Meier method (*P* < 0.001) (Fig. 6A). The independent risk factors “multiple lesions” and “ASI”, were significantly different in the Kaplan–Meier single factor survival analysis (Fig. 6B and C).

## Discussion

PEM is a rare kind of endometriosis, and accounts for 0.29% (130/44828, Fig. 1) of all kinds of endometriosis according to our data. Many theories are advocated to explain the occurrence of ectopic endometrium in perineal area. Iatrogenic implantation of endometrial tissue into the open wound at perineum has been proposed as the predominant theory in relation to the genesis of PEM [11]. A recent systemic review incorporating 90 studies found that 95.3% of 283 patients with vulvo-perineal endometriosis had gone through perineal trauma before the onset of symptoms [5]. The results of our survey further corroborate the hypothesis of iatrogenic seeding through wound contact with viable endometrial cells. Nonetheless, spontaneous PEM without perineal injury has been documented as well. They mostly occur in the bartholin gland and labia while seldom involve the perianal muscle [5], which might be explained by coelomic metaplasia, lymphatic and/or haematogenous dissemination, and growth differentiation of bone marrow-derived stem cells (BMDSCs) [12, 13]. BMDSCs may be a source of extra-pelvic endometriosis because of their ability to differentiate directly into endometriotic cells at ectopic sites after migration through peripheral circulation [12]. Also, growing evidence has found that abnormal epigenetic expression [12], microbiome and metabolic changes [14, 15], unbalanced immune microenvironment [16] may involve the development of endometriosis by modulating the proliferation, invasiveness and adhesion of ectopic endometrial cells. However, whether they influence the pathogenesis of PEM remains to be explored.

Despite early management is recommended to prevent the infiltrative growth into adjacent structures, accurate diagnosis of PEM at initial stage is challenging in clinical practise. Zhu et al. suggested three typical characteristics for clinical diagnosis with a high predictive value, including anamnesis of perineal tears and/or episiotomy, tender nodules or masses at perineal scar, and cyclic nature of painful complaint associated with menses [6]. However, the highly variable presentations make PEM easily to be confused with anal abscess, bartholinitis, lipoma, lymphoma, haemangioma and vulvar carcinoma, particularly those women who were transferred to general surgeons at first [5, 17–20]. Hence, clinical practitioners should realise that any cyclical symptom reported by women of reproductive age can be suspected as an indicator of endometriotic lesion.

Consistent to previous findings, serum CA-125 does not assist in PEM diagnosis in our survey since the majority of participants showed normal range concentrations [21]. Ultrasonography has been proposed as a non-invasive, reproducible and cost-effective imaging modality for visualising the lesion diameters, localisation, proximity to neighbouring structures and extent of muscular invasion if present [22]. The sonographic features are usually characterised by hypoechoic solid or cystic nodules containing hyperechoic spots or bright strands with ill-defined borders at the site of episiotomy scar [3]. Peripheral vascularisation may be revealed through Doppler evaluation [3, 6]. Endoanal ultrasonography is a reliable technique to distinguish the perianal abnormalities and to delineate the structural integrity of anal sphincter with high accuracy, through which sphincter or rectal involvement can be easily determined [23]. As a complementary method to endo-sonography, trans-perineal ultrasonography has been deemed as a more widely available option to measure the size of perianal lesion and to assess its anatomic relationship to vital structures [4]. Pelvic MRI has superb contrast resolution for soft tissues and is used to identify very small lesions or deep endometriotic tissue with extensive infiltration. PEM lesions characteristically appear as hyperintense heterogenous spots on T1-weighted images and hypointense nodules on T2-weighted images due to the periodic hemorrhage inside the ectopic foci [3]. Infiltrative PEM lesions penetrating the mucous membrane of anal or rectal wall could lead to severe intestinal symptoms including painful defecation, rectal bleeding, and chronic diarrhoea. In that case, endoscopic examination such as colonoscopy or proctoscopy should be performed for further investigation.

Despite the very low incidence of extra-pelvic endometriosis, treatment for this ectopic pathology is one of the most challenging issues in clinical practise. Generally, the therapeutic strategy varies with the disease location and symptomatic severity. Medical management of hormone deprivation could greatly relieve the pain whereas surgical removal of the endometrial implants has been viewed as a more radical approach to restore the anatomy and organ function with a pronounced long-term benefit. In regard to the age, infiltrating depth, tumour size and the adjacent structures, different surgical techniques sparing the critical nerve, vessels and important organs should be applied for a minimally invasive treatment with the best effect [24, 25].

As the primary therapeutic modality for symptomatic patients with PEM, surgical treatment is recommended. Local complete excision is associated with lower recurrence and can avoid the possibility of malignant degeneration even though it may partially compromise sphincter muscles. Hormone suppressive therapy with GnRH-a before the surgery is effective in reducing the size of lesions, particularly for lesions adherent to or extending into perianal muscle [26]. There were nearly one third of PEM patients affected with ASI in our series, which was similar to the findings of Liu et al. [21]. Rectal examination should be performed routinely, especially in the case of perianal involvement, to assess the extent of ASI. Accordingly, we detected a positive relation between ASI and recurrence because of increased difficulty in the process of radical removal. Since the majority of cases with ASI experienced no recurrence, radical removal of involved sphincter muscle with sphincteroplasty is preferred as a safe and curative procedure minimising the risk of recurrence [4, 21].

Conceivably, the recurrent endometriotic lesions may arise from minimal residual lesions (MRLs) or from dae novo lesions [27]. Nonetheless, a mounting evidence suggests that the former is more likely. Complete excision of the lesions together with surrounding healthy tissue to make sure that no residual disease left behind was advocated by most practitioners [5]. In the present study, the high incidence of mPM and its potential effect of facilitating post-operative recurrence implied that, merely under visual inspection, MRLs could hardly be avoided in the case of narrow excision with 0.2–0.5 cm of surgical margins. In fact, some researchers have proposed that complete resection required at least 0.5–1.0 cm free edges from the PEM nodules, and even wide excision with 1.0–2.0 cm of peripheral tissue has been also recommended [5]. A recent systemic review of vulvo-perineal endometriosis pointed that wide complete excision produced a lower overall recurrence rate than a mix of all kinds of excision [5].

Minimal residual of PEM and its potential effect on recurrence was discovered in our study for the first time. Based on much larger sample size with longer follow-up period, the rate of local recurrence in our study was significantly higher than previously reported. Surprisingly, late recurrence beyond two years accounts for 43.8%, indicating a continuous surveillance on PEM patients after surgical treatment is recommended. In the circumstances of bowel endometriosis, the impact of mPM on clinical outcome remains inconclusive. Nirgianakis aet al. proposed that positive margins in segmental bowel resection might predict higher recurrence (HR = 6.5, 95% CI 1.8–23.5, *P* = 0.005) [28]. Notwithstanding that Roman aet al. found mPM had no impact on postoperative pain and digestive function [29]. Further investigation of prospective study is required for a better understanding of clinical significance of mPM.

In our study, hormonal medication did not seem to interfere with the risk of recurrence in a statistical significance way, which deviates from the rudimentary results that GnRH-a correlated with reduced recurrence in prior observations. However, after the diagnosis of recurrence, the pain symptom of most recurrent cases were well relieved after timely hormone intervention. Likewise, it has been reported that PEM lesions could spontaneously regress after pregnancy, suggesting the hormone-responsive feature of the disease [30]. In agreement to our findings, Seong et al. found hormone therapy was associated with longer recurrence-free interval from the time of surgery to the onset of recurrence after primary surgery for ovarian endometrioma [31]. Hormonal therapy maintains the minimal disease state by slowing down the regrowth rather than eliminate residuals, as revealed by Sharpe et al. in a rat model that the implant lesion was significantly inhibited by GnRH-a while regrowth sustained spontaneously after the cessation of hormone suppressive treatment []. Taken together, these results suggest postoperative hormonal suppression has beneficial effects on extending disease-free interval but does not completely prevent recurrence of PEM.

To our best knowledge, the present study is unique in that it includes the largest number of consecutive patients with PEM to date and conducts a profound investigation into clinical characteristics, treatment modalities and postsurgical outcomes of this rare entity. Moreover, the postoperative recurrence nomogram of PEM was established accordingly. However, there are still some limits in the present study. Firstly, the follow-up was completed in a manner of outpatient visit and telephone enquiry. There may be recall bias about the exact time of recurrence. Secondly, it’s limited that we took symptomatic recurrence as primary outcomes on this survey because treatment was mainly dominated by symptomatic recurrence. There’s only 37.5% (5/16) of patients with recrudescence of symptoms had histopathologic confirmation which can produce more convincing evidence. Thirdly, the 20% of non-respondent rate may cause some uncertainty on the interpretation of results. We lost in touch with them because of invalid contact information so that their current situation cannot be obtained. Finally, due to the small number of cases, the effectiveness of this nomogram was achieved through internal verification. Therefore, it is still necessary to use external data for further verification in the future.

## Conclusions

Taken together, PEM is a rare kind of endometriosis and often secondary to episiotomy. Patients usually complain tender nodules or masses at perineal scar and cyclic pain associated with menses. Medical history, ultrasound and MRI examination are helpful for diagnosis. Complete excision is considered as the primary treatment of PEM. Multiple lesions and ASI are independent risk factors for postoperative recurrence, and wide resection of endometriotic lesions together with more peripheral tissue could be preferred for recurrence prevention. We developed a predictive nomogram to predict the probability of no recurrence within 36, 60 and 120 months of PEM patients after surgery. The proposed nomogram provided better discrimination with statistical significance and offered a useful tool for prognosis. In addition, to generalise the use of this nomogram in other groups, additional validation with external data is required.

## Data Availability

The article does not involve sequencing data. Demographic information and basic clinical information are already shown in Tables 1 and 2. Other clinical data used in the study are available from the corresponding author upon reasonable request.

## Abbreviations

PEM: Perineal endometriosis
PUMCH: Peking union medical college hospital
SD: Standard deviation
IQR: Interquartile range
C-index: Index of concordance
AUC: Area under the curve
CA-125: Cancer antigen 125
TVS: Transvaginal ultrasound
MRI: Magnetic resonance imaging
GnRH-a: Gonadotrophin-releasing hormone agonist
COCs: Combined oral contraceptives
LNG-IUS: Levonorgestrel-releasing intrauterine system
ASI: Anal sphincter involvement
mPM: Microscopically positive margin
HR: Hazard ratio
MRLs: Minimal residual lesions
