# Correction: Clinical presentation of perineal endometriosis and prognostic nomogram after surgical resection

**DOI:** 10.1186/s12905-022-02100-6

**Published:** 2022-12-06

**Authors:** Shiyang Zhu, Zhiyue Gu, Xiaoyan Li, Yi Dai, Jinghua Shi, Jinhua Leng

**Affiliations:** 1 Department of Obstetrics and Gynecology, Peking Union Medical College Hospital, Chinese Academy of Medical Sciences and Peking Union Medical College, No. 1 Shuaifuyuan No. 1, Dongcheng District, Beijing, 100730 China; 2 National Clinical Research Center for Obstetric and Gynecologic Diseases, Beijing, China; 3 Department of Gynecology and Obstetrics, West China Second University Hospital, Sichuan University, Chengdu, China

**Correction: BMC Women’s Health (2022) 22:476** 10.1186/s12905-022-02068-3

Following publication of the original article [1], In this article in text for ref. 32 has been inserted and the following is the text:

In our study, hormonal medication did not seem to interfere with the risk of recurrence in a statistical significance way, which deviates from the rudimentary results that GnRH-a correlated with reduced recurrence in prior observations. However, after the diagnosis of recurrence, the pain symptom of most recurrent cases were well relieved after timely hormone intervention. Likewise, it has been reported that PEM lesions could spontaneously regress after pregnancy, suggesting the hormone-responsive feature of the disease [30]. In agreement to our findings, Seong et al. found hormone therapy was associated with longer recurrence-free interval from the time of surgery to the onset of recurrence after primary surgery for ovarian endometrioma [31]. Hormonal therapy maintains the minimal disease state by slowing down the regrowth rather than eliminate residuals, as revealed by Sharpe et al. in a rat model that the implant lesion was significantly inhibited by GnRH-a while regrowth sustained spontaneously after the cessation of hormone suppressive treatment [32]. Taken together, these results suggest postoperative hormonal suppression has beneficial effects on extending disease-free interval but does not completely prevent recurrence of PEM.

The original article has been corrected.
